# Invisible cervical cancers on MRI: Can the type of histology (SCC versus non-SCC) influence surgical planning?

**DOI:** 10.3389/fonc.2022.996516

**Published:** 2022-12-08

**Authors:** Jungeun Jeon, Byung Kwan Park, Jeong-Won Lee, Chel Hun Choi, Yoo-Young Lee, Tae-Joong Kim, Byoungi-Gie Kim

**Affiliations:** ^1^ Department of Obstetrics and Gynecology, Samsung Medical Center, Sungkyunkwan University School of Medicine, Seoul, Republic of Korea; ^2^ Department of Radiology, Samsung Medical Center, Sungkyunkwan University School of Medicine, Seoul, Republic of Korea

**Keywords:** uterus, cervical cancer, histology, MRI, FIGO staging

## Abstract

**Background:**

Invisible cervical cancers on MRI can indicate less invasive surgery. Cervical cancers consist of squamous cell carcinoma (SCC) and non-SCC, each with different long-term outcomes. It is still unclear if surgical planning should be changed according to the histologic type of cervical cancer when it is not visible on MRI.

**Purpose:**

The purpose of the study was to determine if surgical planning for cervical cancer that is not visible on MRI is influenced by the histologic type.

**Materials and methods:**

Between January 2007 and December 2016, 155 women had Federation of Gynecology and Obstetrics (FIGO) stage 1B1 cervical cancer that was not visible on preoperative MRI. They underwent radical hysterectomies and pelvic lymph node dissections. Among them, 88 and 67 were histologically diagnosed with SCC and non-SCC, respectively. The size of the residual tumor, depth of stromal invasion, parametrial invasion, vaginal invasion, lymphovascular invasion, and lymph node metastasis were compared between these patients using the t-test, Mann–Whitney U test, Chi-squared test, or Fisher’s exact test. The recurrence-free and overall 10-year survival rates were compared between the groups by Kaplan–Meier analysis.

**Results:**

The mean sizes of residual tumors were 8.4 ± 10.4 mm in the SCC group and 12.5 ± 11.9 mm in the non-SCC group (p = 0.024). The mean depth of stromal invasion in the SCC group was 12.4 ± 21.2% (0%–100%), whereas that in the non-SCC group was 22.4 ± 24.4 (0%–93%) (p = 0.016). However, there was no difference in parametrial or vaginal invasion, lymphovascular invasion, or lymph node metastasis (p = 0.504–1.000). The recurrence-free and overall 10-year survival rates were 98.9% (87/88) and 95.5% (64/67) (p = 0.246), and 96.6% (85/88) and 95.5% (64/67) (p = 0.872), respectively.

**Conclusions:**

The non-SCC group tends to have larger residual tumors and a greater depth of stromal invasion than the SCC group, even though neither is visible on MRI. Therefore, meticulous care is necessary for performing parametrectomy in patients with non-SCC cervical cancer.

## Introduction

Previously reported studies showed that postoperative outcomes were good when Federation of Gynecology and Obstetrics (FIGO) stage IB1 cervical cancer was not visible on preoperative magnetic resonance imaging (MRI) (1–3). This cancer has a much lower tumor burden than those visible on MRI. Accordingly, the former has a better prognosis than the latter. However, previous studies did not investigate whether postoperative outcomes differed according to histologic type. Patients with squamous cell carcinoma (SCC) frequently have better long-term outcomes than those without SCC.

Moreover, the tumor conspicuity of non-SCC is not as good as that of SCC, so it cannot be easily determined if non-SCC cervical cancer is visible on MRI (4–6). Minimizing parametrectomy is useful for avoiding postoperative complications (7–14). However, false-positive results for invisible tumors may lead to underestimating the extent of surgical resection needed. As a result, unnecessary additional treatments may follow a minimally invasive hysterectomy.

Thus, we hypothesized that the sizes of postoperative residual tumors differ according to the histologic types of FIGO stage IB1 cervical cancer, even though these are not visible on preoperative MRI. Rare studies have compared the postoperative outcomes of SCC and non-SCC patients. The purpose of this study was to determine if surgical planning for cervical cancer not visible on MRI is influenced by histologic type (SCC versus non-SCC).

## Materials and methods

This study (File No.: 2022-04-030-001) was approved by the Institutional Review Board at Samsung Medical Center and the requirement for informed consent was waived due to the retrospective design.

### Patients

Between January 2007 and December 2016, a total of 747 patients with FIGO IB1 cervical cancer underwent MRI prior to radical hysterectomy. Among them, 52 patients were excluded due to the poor image quality of the MRI examinations. Among the remaining 695 patients, 540 and 155 had visible cancer and invisible cancer, respectively, on preoperative MRI. Finally, 155 patients were included in the study population when they underwent 1.5 T or 3.0 T MRI. Of them, 88 patients were histologically confirmed to have squamous cell carcinoma (SCC) (SCC group). The remaining 67 patients were histologically confirmed to have other cervical cancers (non-SCC group). The medical records of the patients in the SCC group (48.5 ± 12.1 years; 20–81 years) and the non-SCC group (44.4 ± 8.5 years; 29–64 years) were reviewed. Colposcopic biopsy and conization were performed in 80.0% (124/155) and 60.0% (93/155), respectively.

Bimanual pelvic and rectovaginal examinations were done to determine the disease extent. Laboratory tests, chest radiography, cystoscopy, and sigmoidoscopy were routinely performed for clinical FIGO staging (15). The time interval between MRI and hysterectomy ranged from 1 to 47 days (median, 16 days) in the SCC group and from 0 to 39 days (median, 15 days) in the non-SCC group.

The MR images were preoperatively interpreted by one of two radiologists who had approximately five or more years of experience in gynecologic imaging. They were additionally reviewed by one radiologist who had approximately 19 years of experience in gynecologic imaging.

Radical hysterectomy, vaginectomy, and lymph node (LN) dissection were performed on all patients. Additional surgical procedures depend on the clinical stage and the surgeon’s decision. When pelvic LNs were suspicious for metastasis at frozen sectioning, the para-aortic LNs were dissected.

Two pathologists examined the surgical specimens. They recorded the size of the residual tumor, histologic type, depth of stromal invasion, lymphovascular space (LVS) invasion, parametrial invasion, vaginal invasion, resection tumor margin, and LN metastasis.

After primary treatment, all patients received adequate follow-up procedures. During this period, patients underwent physical examinations, Pap smears, and tumor marker analysis every three months for the first two years and every six months for the next three years. Imaging studies, such as abdominopelvic computed tomography (CT) or pelvic MRI, were conducted every 6 – 12 months for the first two years and then annually for the next three years.

### MR imaging

Pelvic scans were conducted with a 1.5 (n = 27) MRI scanner (Signa, GE Medical System, Milwaukee, USA) or 3 T (n = 128) MRI scanner (Intera Achiva 3T; Philips Medical System, Best, The Netherlands). The upper abdomen was scanned by MRI or CT. The 1.5 T MRI sequences of the pelvis included T2-weighted images (T2WI), T1-weighted images, and dynamic contrast-enhanced (DCE) images. Diffusion-weighted imaging (DWI) was added to the 3 T MRI examination. However, DWI could not be scanned at the 1.5 T MRI because the MR software did not have the capability. T2WI were obtained in the axial, sagittal, and coronal planes. The other sequences were obtained in the axial plane. The upper abdomen was scanned from the lower lung to the aortic bifurcation. The same MR parameters as those used by Park et al. were used (1).

### Data analysis

**Figure 1:**
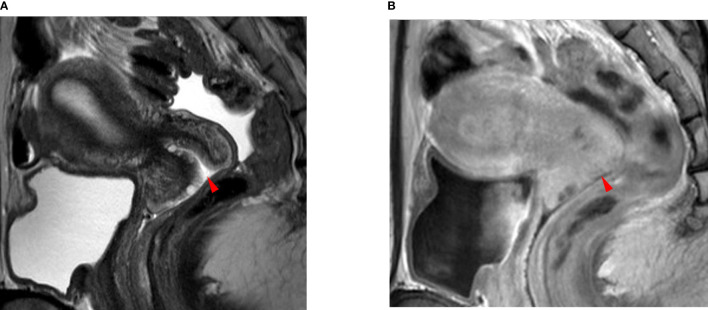
A 35-year-old woman with squamous cell carcinoma. **(A)** The T2-weighted sagittal MR image shows no focal lesion in the cervix. The red arrowhead indicates the external OS of the uterine cervix. **(B)** The delayed contrast-enhanced sagittal MR image shows no residual cancer in the cervix. The red arrowhead indicates the external OS of the uterine cervix. The pathologic report confirmed no residual cancer in the resected uterus. There was also no invasion of the lymphovascular space, vagina, parametrium, or lymph node metastasis.

**Figure 2:**
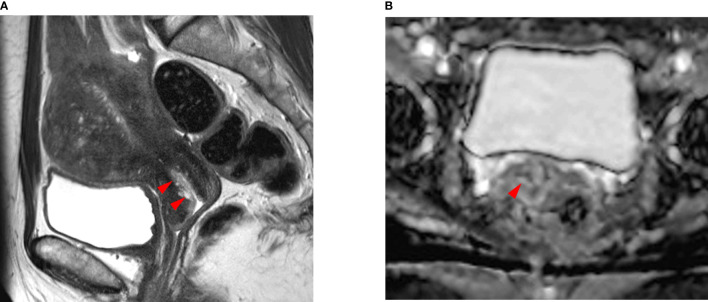
A 48-year-old woman with endocervical adenocarcinoma. **(A)** The T2-weighted sagittal MR image shows no tumor in the uterine cervix. The red arrows indicate a poorly demarcated cystic mass, which was preoperatively interpreted as normal endocervical glands. **(B)** The apparent diffusion coefficient (ADC) axial image shows no focal lesion with low ADC values in the cervical canal (red arrowhead). However, the pathologic report confirmed that there was a residual tumor in the endocervical canal. The tumor size was measured as 2.0 × 1.5 cm and the depth of stromal invasion was 0.4 cm in a 1.3-cm cervical wall. It was well-correlated with the endocervical lesion in **(A)**. Tumor invasion to the lymphovascular space, vagina, and parametrium and lymph node metastasis were all negative.

Invisible cancer was defined when the cervical tumor was invisible on T2W and DCE 1.5T MR images and when it was invisible on T2WI, DWI, and DCE 3T MR images (**Figures 1, 2**). When post-biopsy inflammation was differentiated from cervical cancer on T2W because both were hypertense, DWI or DCE images were reviewed; the former had no diffusion restriction or showed iso- or higher enhancement compared to neighboring cervical tissue, unlike the latter.

Patient age, biopsy type, histologic type, and SCC antigens or other tumor markers were compared between the SCC and non-SCC groups. The size of the residual tumor, depth of stromal invasion, LVS invasion, parametrial invasion, vaginal invasion, and LN metastasis were also compared between the groups.

Recurrent tumors were assessed on follow-up CT or MR images. Recurrence-free and overall 10-year survival rates were calculated and compared between the SCC and non-SCC groups.

### Statistical analysis

Patient age, the size of the residual tumor, and the depth of stromal invasion were compared by the Mann–Whitney test because these data did not show a Gaussian distribution. SCC antigens were compared between two groups using the t-test.

The proportions of biopsy type, cancer histology, LVS invasion, parametrial invasion, vaginal invasion, LN metastasis, and recurrence rate were compared using the chi-square or Fisher’s exact test.

Odds ratios (ORs) and 95% confidence intervals were calculated using the Woolf approximation. When the value was zero, 0.5 was added to each to make the calculation possible.

Recurrence-free and overall 10-year survival rates were compared using Kaplan–Meier survival curves.

Commercially available SPSS 24.0 software for Windows (SPSS Inc., Chicago, IL, USA) was used for the statistical analyses. A p-value of <0.05 was considered statistically significant.

## Results

The median age of the patients in the SCC group was higher than that in the non-SCC group (p = 0.023) (**Table 1**). In the SCC group, 65.9% (58/88) underwent conization and 80.7% (71/88) had colposcopic biopsies, whereas in the non-SCC group, 52.2% (35/67) underwent conization and 79.1% (53/67) had colposcopic biopsies (p = 0.085 and p *=*0.808, respectively). The histologic diagnoses in the non-SCC group included adenocarcinoma in 89.6% (60/67) and adenosquamous carcinoma in 10.4% (7/67). There was no difference in tumor markers (*p* = 0.296–0.906) between the groups.

**Table 1:** Demographics in patients with IB1 SCC and non-SCC cervical cancers.

	FIGO stage IB1 cervical cancers		P values
	SCC (n=88)	Non-SCC (n=67)	
Age (years)	48.5± 12.1 (20–81)	44.4 ± 8.5 (29–64)	0.023
Conization	58 (65.9%)	35 (52.2%)	0.085
Colposcopic biopsy	71 (80.7%)	53 (79.1%)	0.808
SCC antigen (ng/ml)	1.3 ± 4.9 (0–46)	1.5 ± 2.8 (0–15)	0.869
CA-125	8.1 ± 5.7 (2–20)	13.3 ± 17.0 (0–107)	0.296
CA-19-9	5.9 ± 3.8 (2–10)	7.6 ± 6.1 (0–23)	0.906

SCC, squamous cell carcinoma.

Mann-Whitney test was used to compare age, CA-125.

T-test was used to compare SCC antigen.

Chi-square test was used to compare types of biopsy or histological types of cervical cancers.

Age and SCC antigen were shown as median ± standard deviation (range).

The median size of the residual tumor was 8.4 ± 10.4 mm (0–36 mm) in the SCC group and 12.5 ± 11.9 mm (0–55 mm) in the non-SCC group (p = 0.024) (**Table 2**) (**Figures 1**, **2**). The median depth of stromal invasion was 12.4 ± 21.2% (0–100%) in the SCC group and 22.4 ± 24.4% (0–93%) in the non-SCC group (p = 0.016) (**Figures 1**, **2**). Residual tumors in these groups were detected in 52.1% (50/88) and 68.7% (46/67) (*p* = 0.133), respectively. SCC group (n = 88) underwent conization in 58 (65.9%) who had residual cancer in 27 (46.6%). Non-SCC group (n = 67) underwent conization in 35 (52.2%) who had residual cancer in 16 (45.7%). There was no difference between SCC and non-SCC groups regarding the incidence of residual cancer following conization (*p* = 1.000).

**Table 2:** Pathologic comparison of SCC and non-SCC groups.

	FIGO stage IB1 cervical cancers		P values
	SCC (n=88)	Non-SCC (n=67)	
Size of residual tumor (mm)	8.4 ± 10.4 (0–36)	12.5 ± 11.9 (0–55)	0.024
Depth of stromal invasion (%)	12.4 ± 21.2 (0–100)	22.4 ± 24.4 (0–93)	0.016
No residual tumor	38 (43.2%)	21 (31.3%)	0.133
Lymphovascular invasion	8 (9.2%)	5 (7.5%)	0.701
Parametrial invasion	0 (0.0%)	0 (0.0%)	–
Vaginal invasion	1 (1.1%)	0 (0.0%)	1.000
Lymph node metastasis	2 (2.3%)	0 (0.0%)	0.504

T-test was used to compare size of tumor and depth of stromal invasion.

Chi-square test was used to compare no residual tumor and lymphovascular invasion.

Fisher’s exact test was used to compare vaginal invasion, and lymph node metastasis.

Size of tumor and SCC antigen were shown as median ± standard deviation (range).

Parametrial invasion in the SCC and non-SCC groups was detected at 0% (0/88) and 0% (0/67), respectively. LVS invasion was 9.1% (8/88) in the SCC group and 7.5% (5/67) in the non-SCC group, respectively (p = 0.701). LN metastasis was detected in 2.3% (2/88) and 0% (0/67) of the SCC and non-SCC groups, respectively (p = 0.504). Vaginal invasion was detected in 1.1% (1/88) and 0% (0/67) of the SCC and non-SCC groups, respectively (p = 1.000).

The tumor recurrence rate was 1.1% (1/88) in the SCC group and 4.5% (3/67) in the non-SCC group on follow-up CT or MR images (p = 0.316). The recurrence-free 10-year survival rate in the SCC and non-SCC groups was 98.9% (87/88) and 94.5% (64/67) (p = 0.246), respectively. The overall 10-year survival rate was 96.6% (85/88) and 95.5% (64/67) in the SCC and non-SCC groups, respectively (p = 0.872).

Recurrent tumors had the highest OR, at 4.078 in the SCC group versus the non-SCC group. The other ORs ranged from 0 to 1.665 for residual tumor, LN metastasis, LVS invasion, and vaginal invasion.

## Discussion

Our results showed that the residual tumor size in the SCC group was smaller than that in the non-SCC group, even though none of these tumors were visible on MRI. The depth of stromal invasion in the SCC group was also smaller than that in the non-SCC group.

Currently, MRI is more available for women with cervical cancer because it is more precise for measuring tumor size than a physical examination (16–18). These MR images can be scanned in the axial, sagittal, and coronal planes. Therefore, the greatest tumor diameter and tumor volume are measured more accurately by palpation. Gynecologists inspect the outer tumor surface alone, but not the inner margin, which is well-depicted on MRI. This imaging modality provides precise tumor staging, and thus, it is more sensitive to detecting parametrial invasion or endocervical cancer than visual assessment (16–18). MRI also has the potential to avoid intravenous urography, cystoscopy, and sigmoidoscopy if cervical cancer is in the early stages (19–22). Moreover, current FIGO staging requires metastatic work-up in iliac or paraaortic LNs, which are not palpable (22). T2WI is useful for detecting morphologic changes, such as increased size, round shape, and the obliterated fatty hilum of metastatic LNs (23, 24). DWI is sensitive to changes in the tissue cellularity of metastatic LNs (25, 26). These MRI findings are currently used to determine if there is LN metastasis.

Cervical cancer that is not visible on MRI strongly suggests a lower tumor volume compared to those that are visible on MRI (1–3). Therefore, tumor invasion of the parametrium or vagina is extremely rare in invisible cervical cancer. The likelihood of cervical stromal or lymphovascular space invasion is much lower in cervical cancer that is not visible on MRI. LN, or hematogenous metastasis, is also rare. As a result, the long-term survival of patients with invisible cancer is better than that of patients with cancer visible on MRI. Moreover, additional post-operative treatments, such as radiation therapy or chemotherapy, are rarely necessary for women with invisible cervical cancer. Tumor invisibility on MRI can be a strong indicator of minimally invasive surgery.

Huang et al. reported that DCEI improved the depiction of cervical cancer that was not visible on T2WI and DWI (27). Quantitative analysis of DCEI parameters helps enhance the residual tumor after conization. Unfortunately, our study analyzed DCEI by visual assessment alone. Therefore, DCE-MRI quantitative parameters should be added to exclude the likelihood of residual cancer after conization. Hu et al. reported that radiomics had the potential to additionally detect cervical cancer that is not visible on conventional MRI (28). They demonstrated that analyzing radiomics improved diagnostic performance for detecting residual cancer after biopsy or conization. Xia et al. studied radiomics based on a nomogram to predict pelvic LN metastasis in women with early cervical cancer. They achieved high diagnostic accuracy for detecting preoperative pelvic LN metastasis (29).

Park et al. showed that many residual cancers were detected postoperatively even if the tumors were not visible on conventional MR images (1). They tried to identify useful MRI features to allow for minimally invasive surgery because radical hysterectomy with LN dissection results in serious postoperative morbidities. As such, invisible tumors on conventional MR images alone help gynecologists minimize parametrectomy procedures and reduce the extent of LN dissection.

We also agree with their point of view about the clinical significance of cancer-invisible MRI findings. In their research, almost half of the cases had a residual tumor, whose median size was 5 mm. Their 10-year recurrence-free survival rate was almost 100%. As a result, if small residual tumors are detected with new MRI techniques, the patients may undergo unnecessary radical surgery, which seems to be an excessive treatment. When cervical cancer is invisible on T2WI, DWI, and DCEI with visual assessment alone, these MRI findings can provide a clue for indicating minimally invasive surgery.

SCC cervical cancer tends to manifest as a solid tumor on MRI, and thus, the tumor size is easily measured (30). It is well correlated with the tumor size on the hysterectomy specimen. In contrast, the tumor margin of non-SCC cervical cancer is not easily demarcated on preoperative MRI because a cystic component is frequent (4–6). Therefore, if non-SCC is composed mainly of cysts, it is frequently difficult to differentiate from nabothian cysts. Besides, if a few cancer cells are just lining the surface of cysts, current MRI techniques make it difficult to determine if there is a residual tumor following a biopsy. For these reasons, the size of the residual tumor and the depth of stromal invasion in the non-SCC group could not be easily identified on preoperative MRI. These findings in the non-SCC group tend to be more frequent than in the SCC group, although neither are visible on MRI.

Radical hysterectomy is the standard treatment for FIGO stage IB1 cervical cancer and, subsequently, improves the long-term survival rate. This surgical technique consists of parametrectomy and LN dissection. Accordingly, patients have a higher risk of postoperative complications, such as voiding difficulty (7–9), anorectal dysfunction (10, 11), sexual dissatisfaction (10, 11), and lymphedema (12–14), if parametrectomy or LN dissection becomes aggressive. Therefore, greater attention is being paid to minimally invasive surgery to minimize these postoperative complications. The patients in our cases had a relatively younger median age (less than 50 years) and a higher overall survival rate. Because of the radical hysterectomy procedure, they have a high likelihood of postoperative morbidities for a long period. From this point of view, excessive surgical resection can be avoided in women who have cervical cancer that is not visible on MRI because local invasion or metastasis is histologically negative in almost all cases.

This study had several limitations. First, it was conducted retrospectively. Therefore, the likelihood of selection bias cannot be excluded. Second, the number of 1.5 T MRI examinations was relatively large. Unfortunately, our 1.5 T scanner could not provide DWI sequences because it was an old version. However, a 1.5 T scanner has a lower signal-to-noise ratio than a 3 T scanner. Third, the number of SCC cases was relatively small, and the proportion of SCC cases was relatively less than that of non-SCC. There was no difference in long-term survival rates, even though the recurrence rate of SCC was not the same as that of non-SCC.

## Conclusion

The non-SCC group tends to have a larger size of residual tumor and a deeper depth of stromal invasion than the SCC group. Despite these histologic results, non-SCC cervical cancer is frequently invisible on preoperative MRI. Therefore, the extent of parametrectomy for non-SCC cervical cancer should be different from that for SCC cervical cancer, even though these tumors are not visible on preoperative MRI.

## Data availability statement

The raw data supporting the conclusions of this article will be made available by the authors, without undue reservation.

## Ethics statement

The studies involving human participants were reviewed and approved by Institutional Review Board of Samsung Medical Center. Written informed consent for participation was not required for this study in accordance with the national legislation and the institutional requirements.

## Author contributions

Conceptualization, JJ, BP, and J-WL. Methodology, JJ, BP, and J-WL. Software, BP. Validation, BP and J-WL. Formal analysis, JJ and BP. Investigation, JJ and BP. Resources, BP. Data curation, JJ and BP. Writing-original draft preparation, JJ and BP. Writing-review and editing, all authors. Visualization, BP. Supervision, BP and J-WL. Project administration, BP. All authors contributed to the article and approved the submitted version.
